# The MeSH heading “Call Center” is due for an update: why we recommend the more precise heading “Emergency Medical Communication Center”

**DOI:** 10.1186/s13049-023-01155-0

**Published:** 2023-12-04

**Authors:** Astrid K. V. Harring, Jesper Blinkenberg, Guttorm Brattebø, Magnus Hjortdahl, Siri Idland, Emil Iversen, Ann-Chatrin. L. Leonardsen, Erik Zakariassen, Trine M. Jørgensen

**Affiliations:** 1 Oslo Metropolitan University, PB 4, St. Olavs plass, Oslo, 0130 Norway; 2 National Centre for Emergency Primary Health Care, NORCE Norwegian Research Centre AS, Bergen, Norway; 3 Department of Clinical Medicine, Faculty of Medicine, University of Bergen, Bergen, Norway; 4 Norwegian National Advisory Unit on Emergency Medical Communication (KoKom), Haukeland University Hospital, Bergen, Norway; 5 Department of Anaesthesia and Intensive Care, Haukeland University Hospital, Bergen, Norway; 6 Division of Prehospital Services, Oslo University Hospital, Oslo, Norway; 7 Department of Anesthesiology and Intensive Care, Østfold Hospital Trust, Østfold, Norway; 8 Department of Global Public Health and Primary Care, University of Bergen, Bergen, Norway

**Keywords:** Cardiopulmonary resuscitation, Emergency medical services, Out of hospital Cardiac Arrest, Paramedicine

## Abstract

Call centers can be found in various industries. However as a Medical Subject Heading (MeSH) the term “Call centers” does not reflect the critical purpose of handling emergency calls. We recommend “emergency medical communication center(s)”, as this provides clarity and precision regarding the primary function and purpose of the center.

## Background

The Medical Subject Headings (MeSH) tree-structure “Emergency Medical Services [N02.421.297]” includes the MeSH Heading “Call Centers” (Tree Number: N02.421.297.036, Unique ID: D000070456). The Scope Note defines it as “*A facility set up for the purpose of handling large volumes of telephone calls. Call Centers typically utilize some form of computer automation for receiving, dispatching, screening, logging and forwarding telephone calls*”. It has been an established Public MeSH since 2017. However, call centers can be found in various industries such as customer service, telemarketing, technical support, sales, health insurance and more [1]. Hence, the term “Call Center” itself does not specify the nature or purpose of the calls being handled.

### Emergency Medical calls

Because of the MeSH headings’ linkage to the tree-structure “Emergency Medical Services” (EMS), we advocate that “Call Center(s)” is vague and due for an update. Worldwide, in cases of medical emergency, Emergency Medical Communication Centers (EMCCs) are the primary contact point between callers and the healthcare system. The EMCCs are manned with personnel responsible for answering emergency calls, assessing and triaging patients over the phone, providing first aid instructions and medical advice, prioritizing necessary resources, dispatching ambulances, and coordinating emergency response services [2]. Consequently, we propose this more specific and descriptive term to replace the previously mentioned “Call Center” term as a MeSH heading.

Preferably, the Scope Note should entail examples of the different emergency medical numbers internationally, such as 9-1-1 in the US, 1-1-2 in most of Europe, as well as other national variations.

### Overlapping headings?

One might discuss the overlap and consequential need for the existing MeSH heading “Emergency Medical Dispatch [N02.421.297.043]” (EMD), which is defined as “the mobilization of emergency care to the locations and people that need them”. In our opinion, this as an operator role or function within the EMCC. However, we recognize that EMDs may be structured differently [3], as there is a large diversity in the organisation of EMDs between countries and, in some cases, within countries [4].

Another existing MeSH heading, “Emergency Medical Service Communication Systems [N02.421.297.058]”, concerns the more technical use of communication systems and should not be confused with emergency care information systems named “information systems” in the MeSH tree-structure.

Our suggestion for the updated heading is:

**Table:** 

Full MeSH Heading: Emergency Medical Communication Center(s)
Short form: EMCC
Scope Note: A facility set up for the purpose of handling emergency medical telephone calls to emergency medical numbers, such as 9-1-1 in the US, 1-1-2 in most of Europe, as well as several national variations. Emergency Medical Communication typically utilizes some form of computer automation and/or assistance for receiving, logging, and forwarding large volumes of emergency telephone calls. Operators assess and triage emergency medical calls, prioritize initiatives, provide medical advice over the phone and dispatch and coordinate Emergency Medical Services response.

### Summary

Using the term “Emergency Medical Communication Center(s)” provides clarity and precision regarding the primary function and purpose of the center. There is a slight variation in the literature, as both the term “center” and “centre” is used regarding EMCC, depending on whether the author uses US or UK English. As the plural form for both are “centers”, and MeSH is published by the National Library of Medicine, center(s) would be the preferred option. By having an updated and specific MeSH, it becomes easier to distinguish EMCCs from other types of call centers, as it provides coherence for researchers and facilitates the availability of relevant knowledge in literature searches.

## Data Availability

Not applicable.

## Abbreviations

EMCC: Emergency Medical Communication Center
EMD: Emergency Medical Dispatch
MeSH: Medical Subject Headings
EMS: Emergency Medical Services
